# Skin‐Lightening Efficacy of Leatherleaf Fern Extract and a Leatherleaf Fern‐Based Facial Serum In Vitro and Clinical Trial

**DOI:** 10.1111/jocd.70844

**Published:** 2026-04-07

**Authors:** Jung‐Wook Kang, Ji‐Won Hur, A‐Reum Jang, Hang‐Eui Cho, In‐Chul Lee

**Affiliations:** ^1^ College of Fusion and Convergence Seowon University Republic of Korea; ^2^ College of Pharmacy, Research Institute of Pharmaceutical Sciences Kyungpook National University Daegu Republic of Korea; ^3^ Creative Innovation Researcher Center Cosmecca Korea co. Ltd. Republic of Korea; ^4^ Department of Cosmetic Science and Technology Seowon University Republic of Korea

**Keywords:** clinical, cosmetic, facial serum, leatherleaf fern extracts, skin‐lightening

## Abstract

**Background:**

Hyperpigmentation, caused by excessive melanin accumulation, is a significant cosmetic concern in dermatology. Natural compounds are receiving attention as safe and effective skin‐lightening agents due to their antioxidant properties and ability to inhibit melanin synthesis.

**Aims:**

To investigate the antioxidant and melanin biosynthesis inhibitory effects of leatherleaf fern water extract (LFWE) and the skin‐lightening efficacy of a facial serum containing LFWE through in vitro and clinical trials:

**Materials and Methods:**

The antioxidant and tyrosinase inhibitory activities of LFWE were confirmed in vitro, along with its inhibitory effects on factors involved in melanin biosynthesis. Its skin‐lightening effects were then validated through clinical trials.

**Results:**

LFWE demonstrated excellent 1,1‐diphenyl‐2‐picrylhydrazyl and ABTS cation radical scavenging activity and inhibited mushroom tyrosinase activity in a concentration‐dependent manner. LFWE also suppressed melanin biosynthesis in B16‐F10 melanoma cells without toxicity up to 500 μg/mL and significantly inhibited the protein expression levels of tyrosinase, TRP‐1, and TRP‐2, which promote melanin production. Clinical trials of a serum containing LFWE showed that it reduced the melanin index after 4 and 8 weeks, thereby confirming its skin‐lightening effects.

**Conclusion:**

LFWE exerts excellent antioxidant effects and inhibits the expression of factors involved in melanin biosynthesis, thereby demonstrating skin‐lightening effects. Moreover, LFWE‐containing serum significantly reduced melanin indices in clinical trials.

## 1 Introduction

The epidermis is the outermost layer of the skin and plays a vital role in determining skin color. The skin color is primarily determined by the accumulation of melanin, a natural pigment present in corneocytes within the epidermis. However, exposure to ultraviolet (UV) rays or sun damage can result in a common skin condition known as hyperpigmentation, where certain areas of exposed skin become darker [1]. This occurs due to the overexpression of melanin, which is recognized as both a pathological and cosmetic concern [1, 2]. Although melanin provides protective benefits against UV rays, its overaccumulation can cause skin problems, such as melasma and freckles, due to increased melanin production or melanocyte activity [3]. Melanin production occurs through melanogenesis [4], a biosynthetic pathway involving five major signaling pathways [5]. Melanogenesis is regulated by external factors such as UV rays, α‐melanocyte‐stimulating hormone (α‐MSH), stem cell factor, and nitric oxide, as well as endogenous factors such as inflammation [6]. Mediators such as α‐MSH are secreted after UV exposure and promote melanogenesis by interacting with receptors on melanocyte cell membranes [7]. Specifically, the melanocortin 1 receptor (MC1R), which binds with α‐MSH, promotes the production of cyclic adenosine monophosphate (cAMP), further driving melanin synthesis [8].

In recent years, natural cosmetic products have been explored as potential sources of novel whitening agents, providing comparable efficacy to that of synthetic options along with reducing cytotoxic risks [9]. Some substances derived from medicinal plants inhibit melanogenesis in vitro, resulting in the identification of various whitening agents of biological origin [10]. These compounds have been historically used in food and medicine, with their safety and therapeutic benefits extensively validated [11]. Among natural sources, the fern genus *Rumohra*, specifically *Rumohra adiantiformis* (leatherleaf fern), excels due to its widespread geographic distribution and morphological consistency [12, 13]. Studies have reported that *R. adiantiformis* exhibits anti‐inflammatory properties in lipopolysaccharide (LPS)‐induced RAW 264.7 cells [14, 15]. Moreover, it has the potential to be used as a biocontrol agent against pathogens due to its antimicrobial activity, which may help in disease prevention [16].

Therefore, this study was conducted to investigate the skin‐lightening effect of leatherleaf fern water extract (LFWE) and its facial serum to confirm their antioxidant and whitening activities, demonstrating their potential for use as functional cosmetic products.

## 2 Materials and Methods

### 2.1 Preparation of the Extract

Leatherleaf fern ( *R. adiantiformis* ) was obtained from Jeju, Korea. The sample was washed and dried at 60°C for > 15 h before extraction. The dried sample was then extracted by stirring with more than 10 volumes of water for 24 h, followed by filtering through a nonwoven fabric to separate the precipitate and supernatant; this process was repeated three times. The extract was then filtered using filter paper, and the solvent was removed by decompression. The resulting LFWE sample was frozen and stored at −20°C until use (yield: 6.3%).

### 2.2 DPPH Assay

The DPPH (1,1‐diphenyl‐2‐picrylhydrazyl) radical scavenging assay was performed according to the method described by Blois et al. with some modifications [17]. After mixing the extract by concentration and the DPPH reagent, the mixture was left for 15 min, and then the absorbance was measured at 517 nm using a microplate reader.

### 2.3 ABTS Cation Radical Scavenging Activity

The antioxidant activity of LFWE was determined using the ABTS cation radical scavenging activity according to the protocol described by Roberta [18]. The ABTS assay working reagent was prepared by reacting 7 mM 2,2‐azino‐bis (3‐ethylbenthiazoline‐6‐sulfonic acid) and 2.45 mM potassium persulfate at room temperature for 15–18 h to form ABTS^+^. Each concentration samples 100 μL and diluted ABTS solution 100 μL at each concentration were added in a 1:1 ratio, and the absorbance was measured at 700 nm.

### 2.4 Tyrosinase Inhibition

The tyrosinase inhibition activity was measured as described by Chan et al. [19]. Briefly, 200 U/mL enzyme (tyrosinase from mushroom) was added to a mixture containing the sample, 67 mM sodium phosphate buffer (pH 6.8), and a substrate solution containing l‐DOPA, after which the mixture was reacted at 37°C for 10 min. DOPA chrome produced during the reaction was measured at 492 nm.

### 2.5 Cell Culture

B16‐F10 melanoma cells were cultured in DMEM containing 10% FBS and 1% penicillin/streptomycin (100 U/mL). The cells were subcultured at 37°C in a 5% CO_2_ incubator and used in the experiment after growing to > 80% confluency.

### 2.6 Cell Viability Assay

Cells (1 × 10^4^/well) were seeded in 96‐well plates and incubated for 24 h, followed by treatment with varying sample concentrations and overnight incubation. Cytotoxicity was evaluated using the 3‐(4,5‐dimethylthiazol‐2‐yl)‐2,5‐diphenyl tetrazolium bromide (MTT) assay, wherein the cells were incubated with MTT for 4 h, dissolved in dimethyl sulfoxide (DMSO), and the absorbance was measured at 540 nm using an ELISA reader.

### 2.7 Measurement of Melanin Content

B16‐F10 cells were seeded at a density of 1 × 10^6^ cells/well in a 100‐mm tissue culture dish and cultured at 37°C in a 5% CO_2_ incubator for 24 h. Next, the cells were treated with 100 nM α‐MSH after 2 h, except for the untreated group, and the sample was treated at various concentrations. After the 24‐h incubation period, the medium was removed, and the cells were disrupted using a lysis buffer. The melanin content was then measured using the pellet obtained by centrifugation of the cell sample at 4°C for 20 min at 13200 rpm. The pellet was dissolved in 1 N NaOH solution (containing 10% DMSO) by vortexing, after which the reaction was performed at 90°C for 1 h, and then the absorbance was measured at 405 nm.

### 2.8 Western Blotting

The expression of tyrosinase, tyrosinase‐related protein‐1 (TRP‐1), tyrosinase‐related protein‐2 (TRP‐2), and Microphthalmia‐associated transcription factor (MITF) was measured in α‐MSH‐stimulated B16‐F10 melanoma cells after treatment with LFWE. The cells were harvested and lysed using radioimmunoprecipitation assay (RIPA) buffer. After protein lysis, a concentration gradient of bicinchoninic acid was used for standardization. Proteins were separated on a 10% sodium dodecyl sulfate (SDS)–polyacrylamide gel (PAG) and then transferred to polyvinylidene difluoride membranes that were incubated overnight with primary antibodies. The resulting blots were washed with Tris‐buffered saline containing 0.1% Tween 20 and incubated with horseradish peroxidase–conjugated secondary antibodies. The protein bands were visualized using enhanced chemiluminescence reagents and quantified using the ChemiDoc Imaging System.

### 2.9 In Vivo Clinical Trial

#### 2.9.1 Study Design

A clinical trial was conducted in compliance with the Good Clinical Practice guidelines and was approved by the Institutional Review Board of the Institute (IRB‐E2408‐001). A total of 23 women (average age 46.35 ± 8.99 years and in good general health) were recruited for this formulation trial to determine whether LFWE causes skin irritation and exerts skin‐lightening effects in an in vivo experiment. The sample size was determined based on guidelines for the efficacy evaluation of functional cosmetics in Korea, which recommend a minimum of 20 valid subjects to ensure statistical reliability [20].

#### 2.9.2 Inclusion and Exclusion Criteria

The inclusion criteria specified healthy women aged 19–59 years with Fitzpatrick skin types II, III, or IV, presenting with facial hyperpigmentation, melasma, or lentigo [21]. All subjects were healthy without any clinical complaints concerning acute or chronic physical diseases, including skin diseases. All participants provided written informed consent after being fully explained about the study protocol.

Subjects were excluded if they met any of the following criteria: (1) pregnancy, possible pregnancy, or lactation; (2) history of photoallergic or photosensitive disorders; (3) use of steroid‐containing topical preparations for skin disease treatment within the past month; (4) participation in a similar study within the previous 6 months; (5) sensitive or hypersensitive skin; (6) presence of skin abnormalities (e.g., scarring, moles, erythema, and telangiectasia) at the test site; or (7) use of similar cosmetics or medications on the test site within 6 months before the study. Furthermore, subjects deemed unsuitable based on the investigator's discretion were excluded.

#### 2.9.3 Randomization, Blinding, and Compliance

Participants were allocated to either the test or control group using a randomization number based on their enrollment order. Table 1 shows a comparison of the detailed compositions of the facial serum containing 1% LFWE and the placebo. The two formulations were prepared using identical base ingredients, differing only in the addition of LFWE, ensuring that neither the investigators nor the subjects could distinguish between the active and placebo products (double‐blind). During the 8‐week study period, the subjects were instructed to avoid direct sunlight and excessive UV exposure to minimize confounding factors affecting skin pigmentation. Compliance was monitored through product use diaries and by weighing the returned containers. The compliance rate was calculated as the percentage of actual usage relative to the required usage. The overall compliance rate among the 23 subjects who completed the trial was > 95%, with no participant falling below the minimum compliance threshold of 80%.

**TABLE 1:** Formulation of test facial serum.

Phase	INCI name	Content (%W/W)	
		Sample	Placebo
A	Disodium EDTA	0.02	0.02
	Hyaluronic Acid	2	2
	Glycerin	3	3
	Butylene Glycol	2	2
	Propanediol	2	2
	Carbomer	0.1	0.1
	Ammonium Acryloyldimethyltaurate/VP Copolymer	0.1	0.1
	D.I. water	To 100	To 100
B	Cetearyl Olivate	3	3
	Sorbitan Olivate	5	5
	Cetyl Ethylhexanoate	0.5	0.5
	Dimethicone	0.1	0.1
C	Tromethamine	5	5
	D.I. water	2	2
D	1,2‐Hexanediol	0.05	0.05
	Ethylhexylglycerin	0.1	0.1
E	Fragrance	1	1
F	LFWE	1	—

Abbreviations: INCI, international nomenclature of cosmetic ingredients; LFWE, Leatherleaf fern water extract.

### 2.10 Skin Melanin Measurement Using Mexameter

We measured the skin‐lightening effects of the LFWE‐containing facial serum using Mexameter MX 18 WL. Mexameter contains a specialized probe that emits light at three wavelengths, and a sensor detects the light reflected from the skin [22]. Melanin levels are evaluated using distinct wavelengths, which exhibit varying absorption rates by the melanin pigments and can be calculated from the quantity of the total emitted light [23]. We examined the change in melanin index at baseline and 4 and 8 weeks on the treated area to confirm instrumental scoring assessment.

### 2.11 Statistical Analysis

Experiments were conducted in triplicate. Data are expressed as mean ± standard deviation. For the in vitro experiment, statistical significance was determined using one‐way analysis of variance followed by Dunnett's post hoc test for multiple comparisons against the control group. For the clinical trial, the changes in melanin index over time were examined using data normality and confirmed using the Shapiro–Wilk test. To compare changes between time points (baseline vs. 4 weeks and baseline vs. 8 weeks), the paired *t*‐test was used for normally distributed data. Statistical significance was defined as *p* < 0.05, and 95% confidence intervals were calculated to ensure statistical reliability. All analyses were conducted using SPSS version 23.0 (IBM Corp., Armonk, NY, USA).

## 3 Results

### 3.1 Effect of LFWE on Radical Scavenging Activity

**FIGURE 1:**
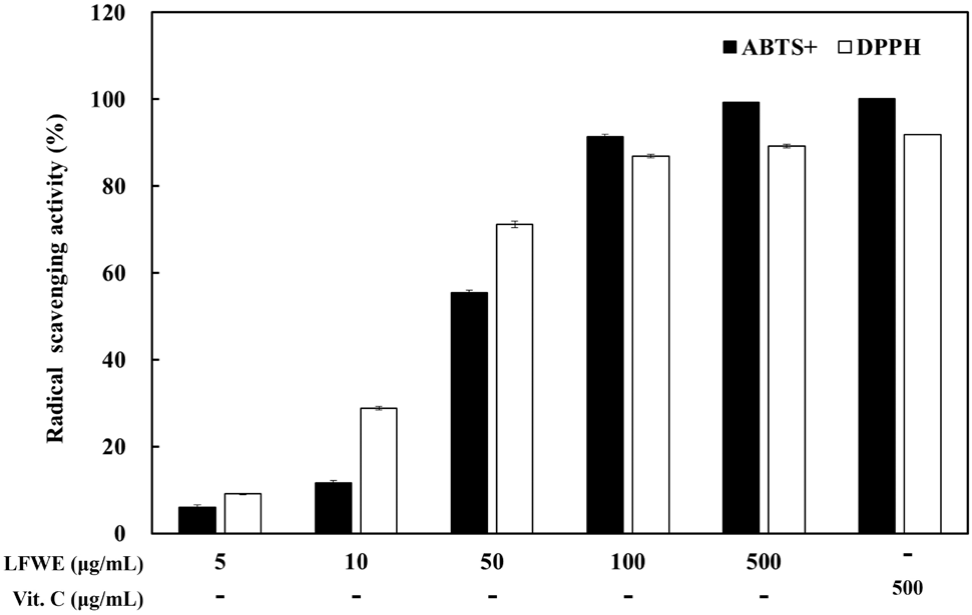
DPPH and ABTS cation radical scavenging effects of LFWE. DPPH radical and ABTS cation radical scavenging activities were measured after reacting with various concentrations of LFWE. Vitamin C (Vit. C, 500 μg/mL) was used as the positive control. Data are expressed as the mean ± standard deviation of three independent experiments. LFWE: Leatherleaf fern water extract.

The antioxidant effects of LFWE were determined using DPPH and ABTS assays. DPPH interacts with electron donors to generate an anionic radical, whereas ABTS produces a cationic radical, resulting in the formation of phenoxy radicals [24]. Treatment with LFWE increased the radical scavenging activity in a dose‐dependent manner in both the DPPH and ABTS^+^ assays (Figure 1). At 500 μg/mL LFWE, the activity was similar to that of the positive control, vitamin C. These results demonstrate that LFWE exerts antioxidant effects to induce radical scavenging activities.

### 3.2 Effect of LFWE on Mushroom Tyrosinase Activity

**FIGURE 2:**
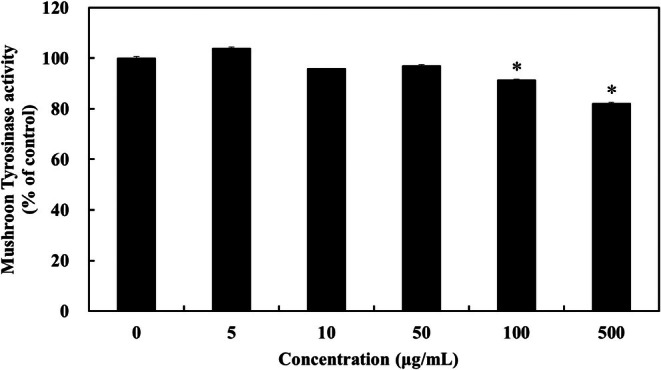
Inhibitory effects of LFWE on mushroom tyrosinase activity. Tyrosinase inhibition was evaluated by mixing LFWE, sodium phosphate buffer, l‐DOPA substrate, and mushroom tyrosinase enzyme. Data are expressed as the mean ± standard deviation of three independent experiments. *p < 0.05 compared with the untreated control group (0 μg/mL).

An in vitro mushroom tyrosinase activity assay was conducted to determine the effects on tyrosinase enzymatic activity. After treating mushroom tyrosinase with various concentrations of LFWE, the tyrosinase activity was significantly inhibited at LFWE concentrations of 100 and 500 μg/mL (Figure 2). These data indicated that LFWE directly affects the tyrosinase enzymatic activity.

### 3.3 Effect of LFWE on Melanin Production in B16‐F10 Cells

**FIGURE 3:**
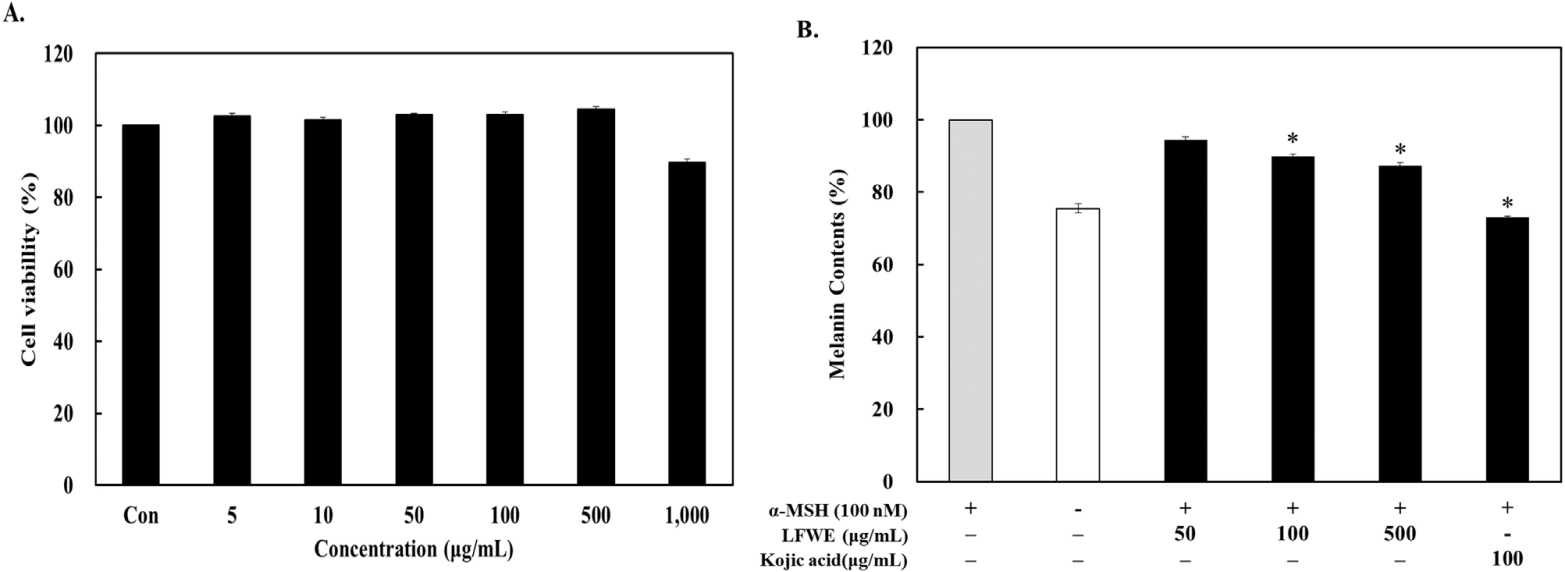
Effects of LFWE on cell viability and melanin biosynthesis in B16‐F10 cells. (A) Cell viability was measured using an MTT assay after treating melanoma cells with various concentrations of LFWE for 24 h. (B) Melanin biosynthesis was measured at 405 nm after treating α‐MSH (100 nM)‐induced B16‐F10 cells with LFWE (50, 100, and 500 μg/mL). Kojic acid (100 μg/mL) was used as a positive control. Data are expressed as the mean ± standard deviation of three independent experiments. *p < 0.05 compared with the group treated with α‐MSH only. LFWE: Leatherleaf fern water extract.

To determine the cytotoxic effect of LFWE, B16‐F10 cells were treated with various concentrations of LFWE for 24 h, after which cytotoxicity in melanoma cells was evaluated using the MTT assay. LFWE exerted no significant effect on cell viability at concentrations up to 500 μg/mL compared with that in untreated controls (Figure 3A). We next measured the melanin content in B16‐F10 cells treated with different concentrations of LFWE (50, 100, and 500 μg/mL). As depicted in Figure 3B, LFWE treatment significantly decreased the melanin content in a dose‐dependent manner. These findings indicate that LFWE decreased the melanin content in α‐MSH‐stimulated B16‐F10 cells.

### 3.4 Effect of LFWE on Melanogenic Enzyme and MITF Activities in B16‐F10 Cells

**FIGURE 4:**
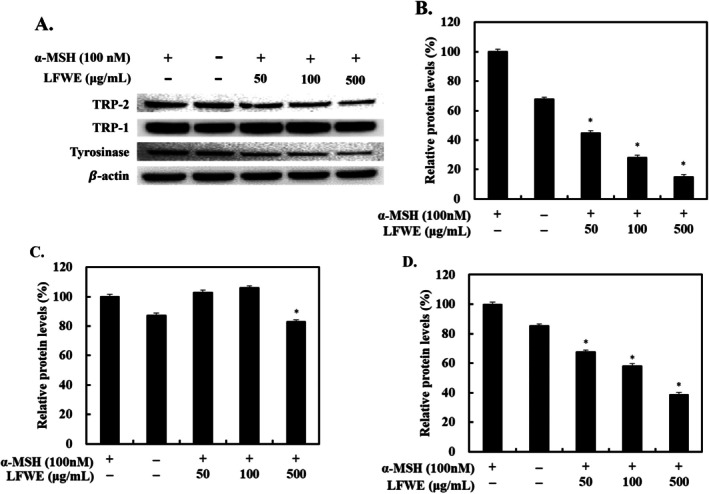
Effects of LFWE on melanogenic enzyme expression in α‐MSH‐stimulated B16‐F10 cells. (A) After a 2‐h pretreatment with α‐MSH (100 nM), cells were treated with LFWE for 24 h. The protein expression of tyrosinase, TRP‐1, and TRP‐2 was analyzed by western blotting, with β‐Actin used as a loading control. Relative protein levels of (B) tyrosinase, (C) TRP‐1, and (D) TRP‐2 were quantified by densitometry. Data are expressed as the mean ± standard deviation of three independent experiments. *p < 0.05 compared with the group treated with α‐MSH only. LFWE: Leatherleaf fern water extract.

Considering that LFWE treatment decreased the melanin content and directly suppressed the tyrosinase activity, we investigated its effects on melanogenic enzymes, tyrosinases, including TRP‐1 and TRP‐2, in α‐MSH‐stimulated B16‐F10 cells. α‐MSH stimulation increased the expression of the melanogenic enzymes in B16‐F10 cells, whereas treatment with various concentrations of LFWE decreased the protein levels of these enzymes (Figure 4). At 500 μg/mL, LFWE exerted the maximum inhibitory effect on melanogenic enzyme expression among the tested concentrations. Next, to explore the upstream regulatory mechanisms, MITF protein levels were examined in B16‐F10 cells. Treatment with 500 μg/mL LFWE resulted in the most significant reduction in MITF expression, decreasing its protein levels by 26.5%. Overall, these data indicate that LFWE effectively inhibits melanogenesis in α‐MSH‐stimulated B16‐F10 cells by downregulating the expression of both the upstream regulator MITF and its downstream melanogenic enzymes.

### 3.5 Skin‐Lightening Effects of LFWE in Clinical Trial

We investigated the skin‐brightening effects of a facial serum containing LFWE in a clinical trial. The melanin index was evaluated at baseline and 4 and 8 weeks using Mexameter., which showed that the facial serum significantly decreased the melanin index compared with that at baseline. In particular, the mean melanin index significantly declined over time (by 2.75% in 4 weeks and 8.01% in 8 weeks) after the application of the LFWE‐containing facial serum. These results suggest that the LFWE‐containing facial serum can improve the skin‐lightening effect in human trials.

## 4 Discussion

Melanin, the primary pigment in skin hyperpigmentation, is often overproduced in localized spots on the skin [25]. Hyperpigmentation is caused by hormones, UV irradiation, and inflammation and plays a role in modulating the immune response of the skin [26]. Excessive accumulation of melanin due to pathological or environmental factors can result in pigmentation disorders, which are widely considered significant aesthetic concerns in dermatology [25]. Skin hyperpigmentation can be treated safely and effectively using various available medications or herbal remedies. Natural products are considered the preferred choice for treating dark spots due to the presence of phenolic components, which demonstrate skin‐lightening properties [27]. We investigated the efficacy of leatherleaf fern extract and facial serum formulations based on this extract and observed skin‐lightening effects caused by the inhibition of melanin production.

Our findings demonstrated that LFWE exhibited radical scavenging activities in a dose‐dependent manner (Figure 1). Moreover, LFWE effectively inhibited the activity of mushroom tyrosinase (Figure 2). As antioxidants and tyrosinase activity are closely associated with the regulation of skin pigmentation, LFWE serves as a critical approach in the development of multifaceted skin‐whitening agents. Resveratrol, one of the most potent antioxidant agents, demonstrates significant tyrosinase inhibitory activity [28]. Various herbs, including *Arachis hypogaea* [4], *Cnidium monnieri* [29], and *Lilium lancifolium* [30], have also been reported to possess antioxidant properties, which are used for the treatment of skin hyperpigmentation.

**FIGURE 5:**
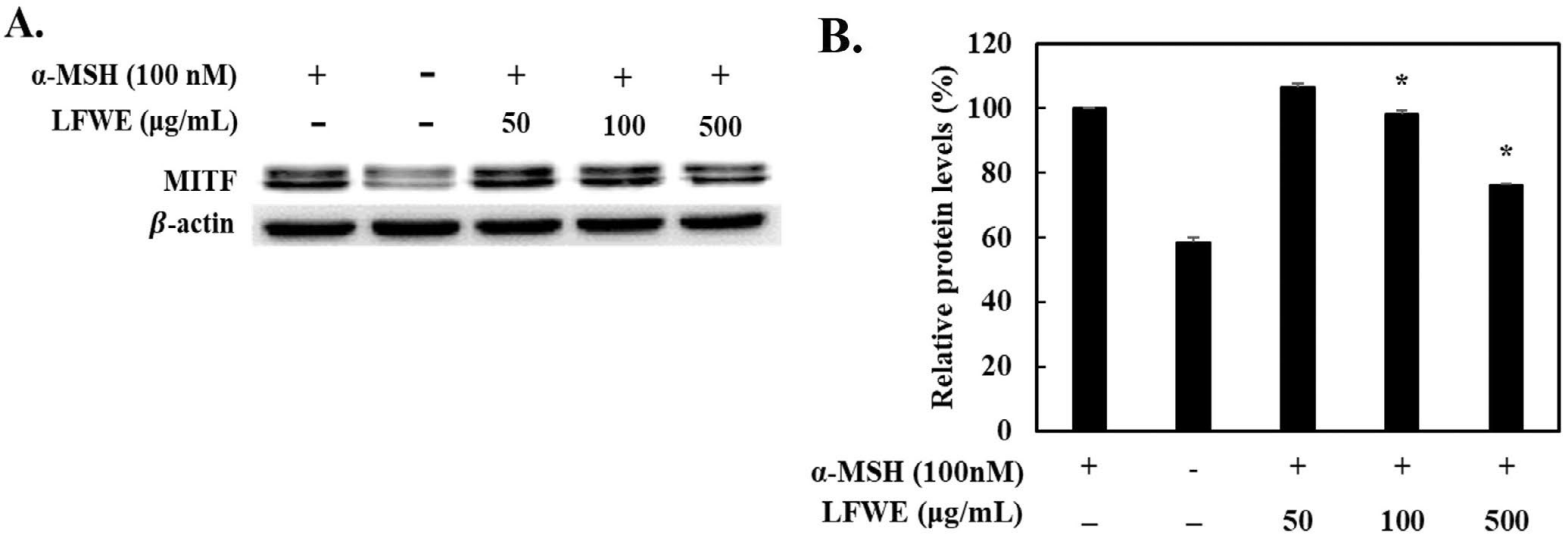
Effects of LFWE on MITF expression in α‐MSH‐stimulated B16‐F10 cells. (A) Representative western blots and (B) relative densitometric quantification of MITF at 24 h after α‐MSH treatment. β‐Actin was used as a loading control. Data are expressed as the mean ± standard deviation of three independent experiments. *p < 0.05 compared with the group treated with α‐MSH only. α‐MSH: α‐Melanocyte‐stimulating hormone; MITF: Microphthalmia‐associated transcription factor. LFWE: Leatherleaf fern water extract.

**FIGURE 6:**
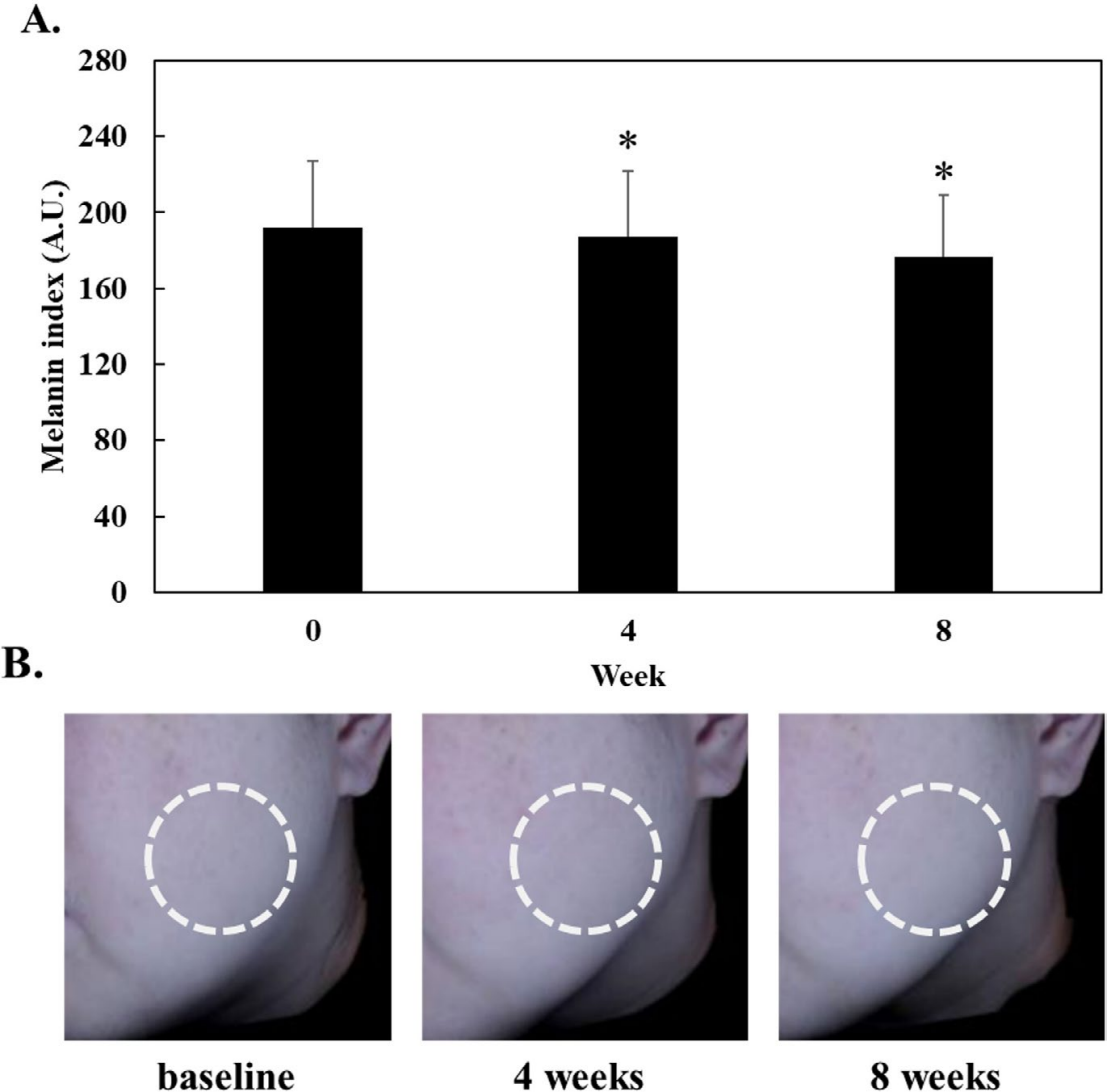
Clinical efficacy of LFWE‐containing facial serum on the skin melanin index. (A) Changes in melanin index at baseline and 4 and 8 weeks after applying the facial serum containing 1% LFWE (*n* = 23, mean age 46.35 ± 8.99 years). Data are expressed as the mean ± standard deviation. *p < 0.05 compared with the baseline (0 weeks). (B) Representative skin images depicting changes in the skin melanin index of a study subject (Subject No. 12) over an 8‐week period.

Melanin synthesis is mediated by tyrosinase, which is produced during the process of melanogenesis. Melanogenesis induces extensive alterations on tyrosinase, which have been identified as melanogenic enzymes, tyrosinase, TRP‐1, and TRP‐2 that upregulate melanogenesis in melanocytes [8]. In the present study, we found that LFWE treatment reduced melanin production in α‐MSH‐stimulated B16‐F10 cells without causing cytotoxicity (Figure 3). Moreover, it decreased the protein levels of tyrosinase, TRP‐1, and TRP‐2 in a dose‐dependent manner in B16‐F10 cells (Figure 4). During melanogenesis, α‐MSH stimulation results in the phosphorylation of PKA and upregulates cAMP levels, which in turn affects CREB signaling and subsequently increases MITF levels in melanocytes [31]. Considering the vital role of MITF in melanogenesis, it serves as a critical target for regulating melanin synthesis [32]. Because LFWE exhibited the maximal efficacy in suppressing the activity of melanogenic enzymes, we further investigated its effect on MITF, a key upstream regulator of melanogenesis, in B16‐F10 cells and found that LFWE treatment significantly decreased the MITF expression (Figure 5). Nevertheless, although it is theoretically well‐established that reducing ROS production can suppress melanin synthesis by downregulating upstream pathways such as cAMP and MITF, our findings regarding the association between antioxidant activity and melanogenesis inhibition establish a phenomenological connection rather than direct molecular evidence [3, 33]. Therefore, to confirm the actual clinical efficacy of LFWE, we further examined the skin‐lightening effect of a facial serum containing 1% LFWE in a clinical trial. We measured the melanin index using a Mexameter, a device used to measure the quantities of major components responsible for skin melanin and hemoglobin [34]. Although LFWE exerted significant antimelanogenic effects at 50–500 μg/mL, topical applications require a higher concentration to effectively overcome the stratum corneum barrier and deliver an active dose to the melanocytes. In pharmacokinetics, it is widely recognized that the stratum corneum acts as a primary barrier, severely limiting the percutaneous absorption of active ingredients [35]. Moreover, incorporating botanical extracts into cosmetic emulsions at concentrations of more than 1%–2% frequently causes formulation instability, such as phase separation, discoloration, and undesirable odors [27]. Our findings confirmed that application of the facial serum significantly reduced the melanin index on the face over an 8‐week period (Figure 6). Similarly, Kuek et al. reported the skin‐lightening properties of creams by quantifying melanin and erythema levels using a Mexameter [22]. Altogether, our results confirmed that LFWE exerts skin‐brightening effects by reducing melanin levels, as demonstrated in both in vitro and clinical trials.

## 5 Conclusion

We investigated the skin‐lightening effect of LFWE and its facial serum by determining their antioxidant and skin‐whitening activities. LFWE demonstrated significant radical scavenging activities and exerted inhibitory effects on tyrosinase expression, resulting in reduced melanin content and suppression of melanogenic enzyme expression in α‐MSH‐stimulated B16‐F10 cells. Furthermore, our clinical trial demonstrated that the facial serum containing LFWE effectively reduced melanin pigmentation. Therefore, LFWE could be a promising therapeutic agent for improving skin brightening in the cosmetic industry.

## Author Contributions

Conceptualization, JW Kang, JW Hur, and AR Jang; methodology, JW Kang, JW Hur, and AR Jang; validation, JW Kang, JW Hur, AR Jang, HE Cho, and IC Lee; formal analysis, JW Kang, JW Hur, AR Jang, HE Cho, and IC Lee; investigation, JW Kang, JW Hur, and AR Jang; data curation, HE Cho and IC Lee; writing – original draft preparation, JW Kang, JW Hur, and AR Jang; writing – review and editing, HE Cho and IC Lee; supervision, HE Cho and IC Lee; project administration, HE Cho and IC Lee. All authors have read and agreed to the published version of the manuscript.

## Ethics Statement

The study protocol was approved by the Institutional Ethics Committee of the K Dermatology Research Center (approval number: IRB‐E2408‐001) in Korea and all procedures adhered to the principles outlined in the Declaration of Helsinki. All participants provided written informed consent prior to enrollment.

## Consent

Informed consent (and an authorization photograph) was obtained from all subjects during inclusion.

## Conflicts of Interest

The authors declare no conflicts of interest.

## Data Availability

The data that support the findings of this study are available from the corresponding author upon reasonable request.
